# Family history of diabetes and glycemic progression: A propensity score-based analysis using health checkup data

**DOI:** 10.1371/journal.pone.0352348

**Published:** 2026-06-24

**Authors:** Sangtaek Oh, Seongtae Kim, Jaesuk Yun, Jung Kee Min, Hee-Jung Jee, Tae-Young Heo

**Affiliations:** 1 Department of Information Statistics, Chungbuk National University, Cheongju-si, Chungcheongbuk-do, Republic of Korea; 2 Department of Mathematics and Statistics, North Carolina A&T State University, Greensboro, North Carolina, United States of America; 3 College of Pharmacy, Chungbuk National University, Cheongju-si, Chungcheongbuk-do, Republic of Korea; 4 Department of Ophthalmology, Ulsan University Hospital, University of Ulsan College of Medicine, Ulsan, Republic of Korea; Japanese Academy of Health and Practice, JAPAN

## Abstract

**Background:**

Family history of diabetes mellitus (FH_ DM) is a well-established risk factor for diabetes, but most studies have focused on disease incidence rather than glycemic changes over time. Understanding how FH_ DM affects the magnitude of change in blood glucose biomarkers before clinical diagnosis can inform earlier intervention strategies and optimize screening intervals.

**Methods:**

We analyzed standardized health checkup data from Korean adults aged 20 years or older with repeated measurements over approximately 2-year (n = 25,647) and 4-year (n = 12,831) intervals. Using propensity score matching (PSM), inverse probability of treatment weighting (IPTW), and doubly robust (DR) estimation, we estimated the average treatment effect of FH_ DM on changes in fasting blood glucose (FBG) and hemoglobin A1c (HbA1c). The analytic cohort included individuals with normoglycemia, prediabetes, and diabetes. Sensitivity analyses using robustness values assessed potential unmeasured confounding.

**Findings:**

After adjusting for 49 measured confounders, individuals with FH_ DM showed consistently greater glycemic progression than those without FH_ DM across all methods. Over 2 years, HbA1c showed a 0.02% greater increase (95% CI: 0.01–0.03) and FBG showed a 0.3 mg/dL greater increase (95% CI: 0.1–0.6) in the FH_ DM group. Over 4 years, the difference in FBG change was 1.3–1.9 mg/dL. Results remained robust when excluding individuals on diabetes medication and across sensitivity analyses.

**Conclusions:**

Family history of diabetes is independently associated with greater glycemic progression across the normoglycemia-to-diabetes spectrum, even after rigorous adjustment for confounding. These findings support the use of FH_ DM as a practical marker for risk-stratified screening strategies. Individuals with FH_ DM may benefit from more frequent glucose monitoring and earlier preventive interventions to delay or prevent diabetes onset.

## Introduction

Diabetes mellitus has become one of the most serious public health challenges in South Korea. The number of individuals diagnosed with diabetes has recently exceeded six million. When those with prediabetes are included, the number of people who either have diabetes or are at risk is estimated to be over twenty million, representing nearly forty percent of the adult population [1]. This heavy burden emphasizes the urgent need for effective strategies that focus on prevention, early detection, and timely intervention. Diabetes can be managed through proper treatment and healthy lifestyle modifications, and the World Health Organization (WHO) has emphasized that early screening for non-communicable diseases is highly cost-effective.

The prevalence of diabetes is rising steadily, which means that the number of people with a family history of diabetes is also expected to increase. Family history is widely recognized as an important risk factor for diabetes. It reflects the combined influence of inherited genetic factors, shared lifestyle and environmental conditions such as diet, exercise, and socioeconomic status, and even possible epigenetic mechanisms [2–6]. Because of these features, family history has long been used as a simple and practical way to identify individuals at higher risk of developing diabetes. At the same time, family history can be subject to reporting errors, and with the growing availability of precision medicine and genetic testing, its role as a proxy measure is becoming more complex.

The diagnosis of diabetes and prediabetes is usually based on fasting blood glucose (FBG) and hemoglobin A1c (HbA1c). These two biomarkers represent different aspects of glucose metabolism. FBG provides information on short-term glucose control and hepatic glucose production, while HbA1c reflects the average blood glucose concentration over the preceding two to three months [7,8]. Examining how family history affects these continuous markers can provide a more detailed understanding of the pathways through which diabetes develops. This type of analysis can also capture early metabolic abnormalities that occur before a clinical diagnosis is made.

Most previous studies have investigated family history in relation to diabetes as a binary outcome [9,10]. Many of these studies have relied on self-reported family history and used cross-sectional regression models or machine learning approaches. Such methods can identify associations, but they may fail to address confounding properly and cannot describe the relationship between family history and glucose regulation in detail. Recent studies have examined family history effects on diabetes complications in diagnosed patients [10], but few have investigated whether family history of diabetes (FH_ DM) affects glycemic progression *before* clinical diagnosis. Understanding the magnitude of glycemic change in individuals with FH_ DM across the normoglycemia-to-diabetes spectrum is critical for informing screening interval decisions and early intervention strategies.

The present study has several strengths that differentiate it from earlier work. First, we use continuous outcomes, namely FBG and HbA1c, rather than relying only on a binary diabetes diagnosis. This enables us to detect differences in glycemic progression that may precede the onset of diabetes. Second, we analyze high-quality laboratory data from standardized national health checkups in South Korea. These checkups provide objective and comprehensive information that includes biochemical and clinical measurements, which reduces the risk of bias from self-reported data [11]. Third, we apply propensity score methods to estimate the average treatment effect of family history, which allows us to approximate the balance achieved in randomized trials and to provide more robust causal estimates. The use of multiple propensity score approaches (including matching, weighting, and doubly robust estimation) has been successfully applied in diabetes research [10,12] and strengthens confidence in our findings.

The aim of this study is to evaluate the effect of family history of diabetes on changes in FBG and HbA1c levels. By focusing on continuous biomarkers and by applying rigorous causal inference methods, this research seeks to clarify whether individuals with a family history of diabetes display different patterns of glycemic progression compared with those without such a history. The findings are expected to contribute evidence that will inform public health policies and support strategies for the early prevention and management of diabetes.

## Materials and methods

### Study design and data source

This retrospective cohort study used data from the clinical data warehouse platform and electronic medical records at Ulsan University Hospital, Republic of Korea. Data were accessed from the institutional database on March 15, 2022, under approval from the Institutional Review Board of Ulsan University Hospital (approval number: UUH 2022-01-031). All data were fully anonymized before analysis, and researchers had no access to information that could identify individual participants during or after data collection. The study was conducted in accordance with the Declaration of Helsinki. Informed consent was waived by the ethics committee due to the retrospective nature of the study and the use of fully anonymized secondary data from routine health checkups.

**Fig 1:**
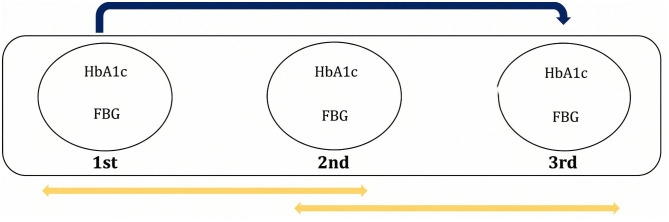
Intervals of hemoglobin A1c and fasting blood glucose measurements across three periods. Each interval is approximately 2 years. The arrow at the top indicates the 4-year difference between the 1st and 3rd visits (Period 2), while the arrows at the bottom denote the 2-year differences between consecutive visits (Period 1).

Participants were adults aged 20 years or older who underwent standardized health checkups. Health checkup data were organized into repeated measurement periods of approximately two years each. We defined glycemic progression as the change in fasting blood glucose (FBG) and hemoglobin A1c (HbA1c) between periods. The difference between the first and second checkups was defined as a one-period (approximately 2-year) change, and the difference between the first and third checkups as a two-period (approximately 4-year) change (Fig 1).

The analytic cohort included individuals with normoglycemia, prediabetes, and diabetes. In Period 1 (2-year interval), 25,647 individuals were included: 3,149 with family history of diabetes (FH_ DM) and 22,498 without FH_ DM. In Period 2 (4-year interval), 12,831 individuals were included: 1,661 with FH_ DM and 11,170 without FH_ DM. To assess robustness, we performed a sensitivity analysis excluding individuals prescribed diabetes medication, which reduced the samples to 25,560 (Period 1) and 12,747 (Period 2) individuals.

### Exposure and outcome definitions

Family history of diabetes mellitus (FH_ DM) was defined as a binary indicator of whether participants self-reported any family history of diabetes in their health checkup records. This served as the exposure variable, with individuals reporting FH_ DM comprising the treated group and those without FH_ DM comprising the untreated group.

The primary outcomes were changes in two blood glucose biomarkers: fasting blood glucose (FBG, measured in mg/dL) and hemoglobin A1c (HbA1c, measured in %). These continuous outcomes represent glycemic progression over time and provide complementary information about glucose metabolism. FBG reflects short-term glucose control and hepatic glucose production, while HbA1c represents average blood glucose concentration over the preceding 2–3 months [7,8]. Because the outcomes were defined as changes from baseline, inter-individual differences in baseline glycemic status are inherently incorporated into the outcome, supporting the validity of the pooled analysis across glycemic strata.

### Covariates

The dataset included 49 baseline covariates measured at the beginning of each analysis interval (Table 1). These comprised: demographic factors (age, sex); anthropometric measurements (body mass index, waist circumference, body fat percentage); vital signs (blood pressure, pulse rate); pulmonary function (forced vital capacity, FEV_1_/FVC ratio); complete blood count parameters; metabolic markers (lipid profile, liver enzymes, kidney function tests, electrolytes); inflammatory markers (ESR, CRP); thyroid function tests; urine analysis; *Helicobacter pylori* status; and medication use for hypertension, diabetes, and hyperlipidemia. Variables irrelevant to glucose metabolism (blood type, urine specific gravity) were excluded from the analysis.

**Table 1:** Baseline characteristics and summary statistics by period. Continuous variables are presented as mean (standard deviation) or median (Q1, Q3). Categorical variables are presented as number (%).

Covariate	Definition	Period 1 (*n* = 25,647)	Period 2 (*n* = 12,831)
Age	Age	50.016 (6.517)	50.843 (6.531)
Sex	Sex (0: male, 1: female)	5,569 (21.7%)	2,788 (21.7%)
BFP	Body Fat Percentage	24.552 (4.860)	24.727 (4.825)
BMI	Body Mass Index	23.977 (2.797)	24.023 (2.812)
WC	Waist Circumference	84.853 (7.343)	85.134 (7.412)
FVC	Forced Vital Capacity	4.226 (0.822)	4.239 (0.830)
FEV_1_/FVC	FEV_1_/FVC ratio	79.052 (5.700)	78.912 (5.632)
SBP	Systolic Blood Pressure	122.854 (11.296)	123.641 (11.231)
DBP	Diastolic Blood Pressure	76.969 (8.624)	76.837 (8.803)
PR	Pulse Rate	72.014 (13.515)	72.439 (14.412)
WBC	White Blood Cell	5.526 (1.510)	5.421 (1.476)
RBC	Red Blood Cell	4.744 (0.408)	4.721 (0.404)
Hct	Hematocrit	44.330 (3.748)	44.153 (3.680)
RDW	Red Cell Distribution Width	13.100 (12.800, 13.500)	13.100 (12.800, 13.500)
PLT	Platelet count	230.565 (49.479)	235.329 (50.696)
Lymphocyte	Lymphocyte	35.157 (7.831)	35.215 (7.826)
Monocytes	Monocyte	7.106 (1.689)	7.147 (1.668)
Eosinophil	Eosinophil	2.300 (1.400, 3.700)	2.300 (1.400, 3.600)
Basophil	Basophil	0.700 (0.500, 0.900)	0.700 (0.500, 0.900)
ESR	Erythrocyte Sedimentation Rate	7 (4, 12)	7 (4, 13)
CRP	C-Reactive Protein	0.05 (0.024, 0.090)	0.05 (0.030, 0.090)
TP	Total Protein	7.041 (0.386)	7.050 (0.395)
ALB	Albumin	4.578 (0.251)	4.586 (0.253)
TBiL	Total Bilirubin	0.888 (0.392)	0.882 (0.388)
DBiL	Direct Bilirubin	0.320 (0.117)	0.319 (0.116)
AST	Aspartate Aminotransferase	21 (17, 25)	21 (18, 26)
ALT	Alanine Transaminase	21 (15, 29)	21 (16, 30)
GGT	Gamma-Glutamyl Transferase	26 (17, 44)	26 (17, 44)
ALP	Alkaline Phosphatase	62.734 (16.306)	63.879 (16.591)
BUN	Blood Urea Nitrogen	13.287 (3.346)	13.363 (3.391)
Cr	Creatinine	0.850 (0.158)	0.858 (0.161)
eGFR	Estimated Glomerular Filtration Rate	98.756 (18.165)	96.386 (14.517)
Uric Acid	Uric Acid	5.484 (1.328)	5.495 (1.332)
TC	Total Cholesterol	191.818 (31.776)	190.627 (31.807)
TG	Triglycerides	102 (71, 148)	103 (73, 151)
HDL	High-density Lipoprotein	53.830 (14.898)	53.735 (14.547)
Ca	Calcium	9.304 (0.337)	9.305 (0.341)
P	Phosphorus	3.270 (0.460)	3.279 (0.466)
Na	Sodium	141.240 (1.912)	141.330 (1.803)
K	Potassium	4.226 (0.295)	4.216 (0.292)
TSH	Thyroid-Stimulating Hormone	1.790 (1.210, 2.660)	1.830 (1.250, 2.730)
Free T_4_	Free Thyroxine	1.284 (0.216)	1.291 (0.225)
T_3_	Triiodothyronine	1.089 (0.216)	1.081 (0.211)
*H. pylori*	*Helicobacter pylori*	2.998 (2.815)	2.897 (2.775)
pH	Urine pH	6.004 (0.708)	5.989 (0.722)
Urine Protein	Urine Protein	1.431 (0.603)	1.496 (0.627)
Med HTN	Medication for Hypertension	395 (1.5%)	380 (3%)
Med DM	Medication for Diabetes Mellitus	87 (0.3%)	84 (0.7%)
Med HLD	Medication for Hyperlipidemia	265 (1%)	243 (1.9%)

### Statistical analysis

#### Analytical framework.

We estimated the average treatment effect (ATE) of FH_ DM on glycemic progression using propensity score methods within a potential outcomes framework [13–16]. Given that family history is a non-manipulable characteristic, these estimates are interpreted as adjusted associations between individuals with and without FH_ DM rather than as effects of a literal intervention. This approach addresses confounding by balancing measured covariates between individuals with and without FH_ DM, thereby approximating the balance achieved in randomized trials.

The ATE represents the expected difference in outcomes if the entire population had FH_ DM versus if they did not. In randomized trials, ATE can be estimated as the simple difference in mean outcomes between groups. However, in observational studies, propensity score methods are needed to adjust for confounding [16,17].

This approach requires several key assumptions [18,19]: (1) no unmeasured confounding after adjusting for observed covariates (conditional exchangeability); (2) both treated and untreated individuals exist across all covariate combinations (positivity); and (3) well-defined treatment with no interference between individuals (Stable Unit Treatment Value Assumption, SUTVA). While these assumptions cannot be definitively verified, we assessed their plausibility through balance diagnostics and sensitivity analyses.

#### Propensity score estimation and methods.

Propensity scores (the conditional probability of having FH_ DM given baseline covariates) were estimated using logistic regression with all 49 covariates measured at the start of each period [17]. We then applied three complementary propensity score methods:

**Propensity score matching (PSM):** We performed 1:1 nearest-neighbor matching without replacement using a caliper width of 0.2 standard deviations of the logit propensity score [20,21]. This caliper approach has been shown to effectively reduce bias while maintaining adequate sample size. Matching created comparable treated and untreated groups, after which we estimated the ATE as the mean difference in outcomes between matched pairs.

**Inverse probability of treatment weighting (IPTW):** Each individual was weighted by the inverse of their probability of receiving their actual treatment status [22,23]. Specifically, treated individuals received weight 1/P(FH_ DM=1|X) and untreated individuals received weight 1/P(FH_ DM=0|X), where *X* represents the covariates. This weighting creates a pseudo-population in which measured confounders are balanced across groups. We then estimated the ATE using weighted linear regression [24].

**Doubly robust (DR) estimation:** This method combines propensity score weighting with outcome regression modeling [25,26]. The DR estimator provides valid results if either the propensity score model or the outcome model is correctly specified, thereby offering additional protection against model misspecification.

#### Balance assessment and sensitivity analysis.

**Fig 2:**
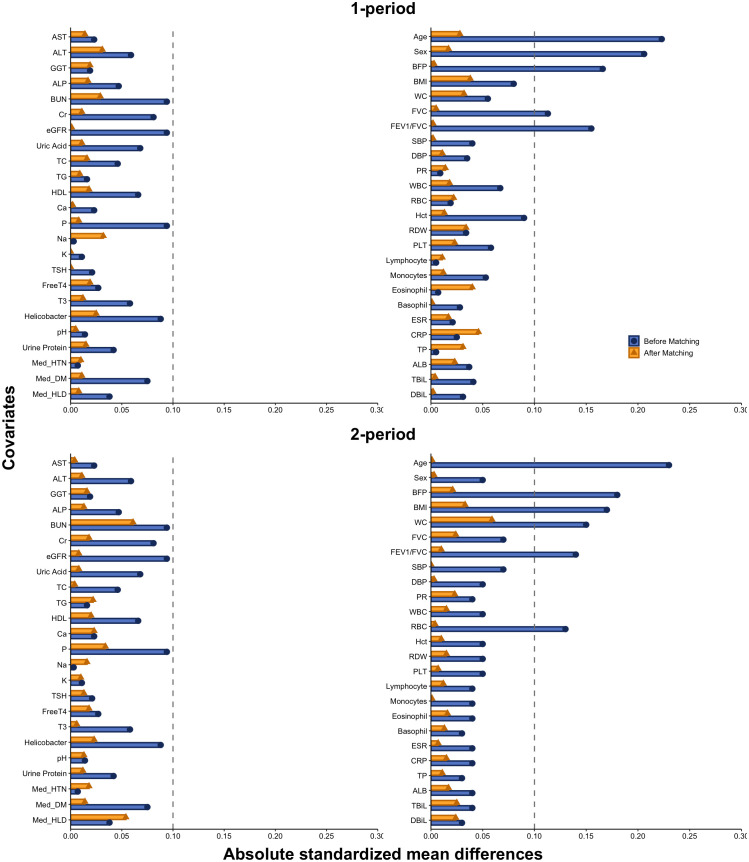
Absolute standardised mean differences between the treated and untreated groups before and after PSM. Vertical dashed lines indicate a standardised mean difference of 0.1.

**Fig 3:**
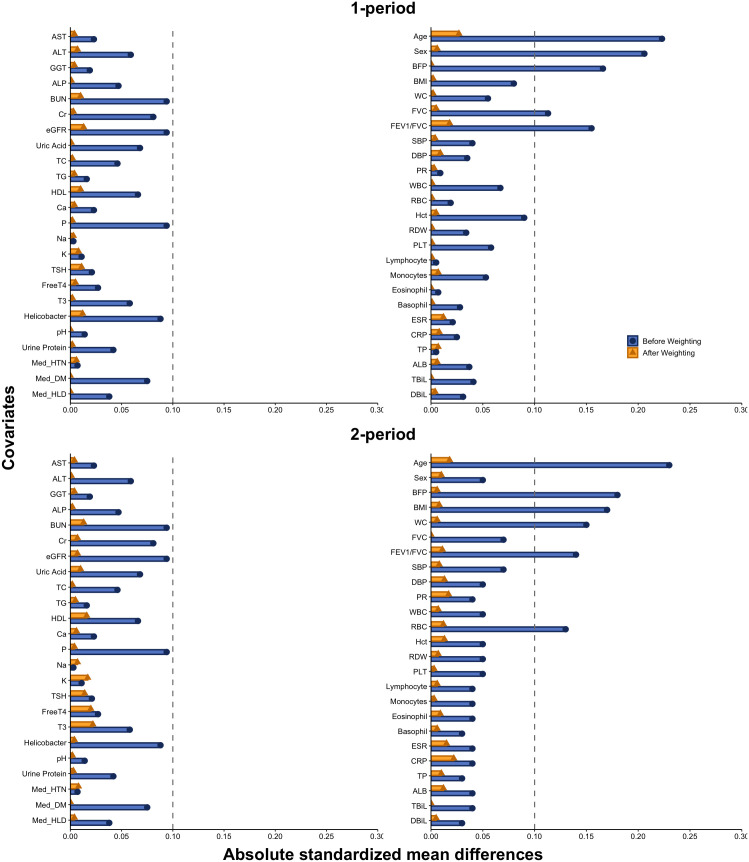
Absolute standardised mean differences between the treated and untreated groups before and after IPTW. Vertical dashed lines indicate a standardised mean difference of 0.1.

We assessed covariate balance using standardized mean differences, defined as the mean difference between groups divided by the pooled standard deviation [27,28]. Standardized differences below 0.1 (10%) were considered to indicate adequate balance. Balance diagnostics are presented in Fig 2 and Fig 3 for PSM and IPTW, respectively.

To evaluate robustness to potential unmeasured confounding, we calculated robustness values (RV) [29]. The RV quantifies the minimum proportion of residual variance that an unmeasured confounder would need to explain in both the exposure and outcome simultaneously to reduce the estimated association to zero. For example, an RV of 0.03 indicates that an unmeasured confounder would need to explain at least 3% of the residual variance in both FH_ DM and glycemic progression simultaneously to nullify our findings. Values below 0.05 suggest potential sensitivity to unmeasured confounding, and results should therefore be interpreted with appropriate caution.

All analyses were conducted using R version 4.3.1. Continuous variables were summarized as mean (SD) for normally distributed data and median (Q1, Q3) for skewed data. Categorical variables were summarized as number (%). Statistical significance was assessed using 95% confidence intervals.

## Results

### Study population and baseline characteristics

A total of 25,647 participants were included in Period 1 (2-year interval) and 12,831 in Period 2 (4-year interval). Among them, 3,149 (12.3%) and 1,661 (12.9%) participants, respectively, reported a family history of diabetes (FH_ DM).

Baseline characteristics are presented in Table 1. Before adjustment, participants with FH_ DM were slightly older and had higher body mass index and waist circumference compared with those without FH_ DM. After applying propensity score methods, all 49 covariates achieved standardized mean differences below 0.1 in both PSM and IPTW, indicating adequate balance between groups (Figs 2 and 3).

### Average treatment effects of family history on glycemic progression

Table 2 presents the estimated average treatment effects (ATE) of FH_ DM on changes in HbA1c and FBG. Results were generally consistent across methods, with IPTW and DR showing similar effect sizes. PSM yielded larger estimates in the 2-period analysis, particularly for FBG, and was therefore interpreted as supportive evidence rather than the primary reference for effect magnitude.

**Table 2:** Average treatment effect of family history on glycemic progression. Values represent the difference in change in HbA1c (%) or FBG (mg/dL) between individuals with family history of diabetes and those without, with 95% confidence intervals in parentheses. PSM, propensity score matching; IPTW, inverse probability of treatment weighting; DR, doubly robust estimation.

Period	PSM	IPTW	DR
**HbA1c (%)**			
1-period (2 years)	0.020 (0.014, 0.026)	0.021 (0.013, 0.030)	0.022 (0.009, 0.035)
2-period (4 years)	0.034 (0.023, 0.045)	0.017 (0.003, 0.031)	0.020 (0.000, 0.042)
**FBG (mg/dL)**			
1-period (2 years)	0.289 (0.090, 0.487)	0.293 (0.013, 0.573)	0.299 (−0.125, 0.757)
2-period (4 years)	1.912 (1.596, 2.227)	1.317 (0.889, 1.745)	1.361 (0.712, 2.079)

**HbA1c changes:** Over 2 years, individuals with FH_ DM showed a 0.02% greater increase in HbA1c compared to those without FH_ DM, with consistent results across all three methods (PSM: 0.020%, 95% CI: 0.014–0.026; IPTW: 0.021%, 95% CI: 0.013–0.030; DR: 0.022%, 95% CI: 0.009–0.035). Over 4 years, IPTW and DR continued to show similar effects (0.017–0.020%), while PSM indicated a larger effect (0.034%, 95% CI: 0.023–0.045).

**FBG changes:** The adjusted differences in FBG were more pronounced and increased substantially over longer follow-up. Over 2 years, the difference in FBG change between groups was approximately 0.3 mg/dL (PSM: 0.289 mg/dL, 95% CI: 0.090–0.487; IPTW: 0.293 mg/dL, 95% CI: 0.013–0.573; DR: 0.299 mg/dL, 95% CI: −0.125–0.757). Over 4 years, this difference increased substantially to 1.3–1.9 mg/dL across methods (PSM: 1.912 mg/dL, 95% CI: 1.596–2.227; IPTW: 1.317 mg/dL, 95% CI: 0.889–1.745; DR: 1.361 mg/dL, 95% CI: 0.712–2.079).

Across both outcomes and time periods, individuals with FH_ DM consistently showed greater glycemic progression than those without FH_ DM. The consistency of findings across IPTW and DR methods, which employ different analytical strategies, strengthens confidence in these results. Of note, the between-group difference in FBG change increased approximately four- to six-fold from the 2-year to the 4-year period, suggesting an acceleration of glycemic divergence over time. In contrast, HbA1c differences remained comparatively stable across periods, reflecting a more gradual and sustained pattern of progression.

### Sensitivity analyses

Excluding individuals on diabetes medication yielded highly similar results (Table 3), confirming that our findings were not driven by pharmacological treatment effects. In this medication-excluded analysis, the pattern of results remained consistent: HbA1c showed differences of 0.016–0.025% and FBG showed differences of 0.343–1.906 mg/dL depending on the period and method used.

**Table 3:** Average treatment effect excluding individuals on diabetes medication. Values represent the difference in change in HbA1c (%) or FBG (mg/dL) between individuals with family history of diabetes and those without, with 95% confidence intervals in parentheses. PSM, propensity score matching; IPTW, inverse probability of treatment weighting; DR, doubly robust estimation.

Period	PSM	IPTW	DR
**HbA1c (%)**			
1-period (2 years)	0.016 (0.010, 0.022)	0.021 (0.012, 0.029)	0.021 (0.008, 0.034)
2-period (4 years)	0.025 (0.015, 0.036)	0.016 (0.003, 0.030)	0.019 (−0.001, 0.042)
**FBG (mg/dL)**			
1-period (2 years)	0.443 (0.244, 0.642)	0.344 (0.069, 0.619)	0.343 (−0.093, 0.813)
2-period (4 years)	1.906 (1.588, 2.223)	1.402 (0.978, 1.826)	1.455 (0.756, 2.168)

Additional stratified analyses by baseline glycemic status were performed to assess whether the pooled analysis was supported across glycemic categories (S1 Table). The positive association between FH_ DM and glycemic progression was generally observed among participants with normoglycemia and prediabetes, particularly for FBG over 4 years. Estimates in the diabetes subgroup were imprecise because of the small sample size, especially in the 4-year analysis.

Robustness value analysis (Table 4) indicated that unmeasured confounders would need to explain 1.1–4.4% of the residual variance in both FH_ DM and the outcomes to nullify our findings. For example, the robustness value for HbA1c in the 2-year analysis was 0.027, meaning an unmeasured confounder would need to explain at least 2.7% of the residual variance in both treatment and outcome to reduce the estimated association to zero. While these relatively low values suggest potential sensitivity to unmeasured confounding, the comprehensive adjustment for 49 measured covariates and consistency of results across multiple methods support the validity of our estimates.

**Table 4:** Robustness values for sensitivity to unmeasured confounding. Robustness value indicates the proportion of residual variance that an unmeasured confounder would need to explain in both treatment and outcome to reduce the estimated association to zero.

Outcome	1-period (2 years)	2-period (4 years)
HbA1c	0.027	0.025
FBG	0.011	0.030

## Discussion

This study provides evidence that family history of diabetes mellitus (FH_ DM) is associated with greater glycemic progression, even after rigorous adjustment for measured confounders. Using multiple propensity score methods applied to high-quality health checkup data, we found that individuals with FH_ DM showed consistently larger increases in both HbA1c and fasting blood glucose compared to those without FH_ DM.

The observed differences in glycemic progression, while modest in absolute terms at the individual level, carry meaningful implications at the population level. While a 0.02% greater increase in HbA1c over 2 years would not in isolation shift a single patient’s clinical management, across a large population of individuals with family history of diabetes, this difference represents a substantial shift in the overall distribution of glycemic risk, with implications for screening policy and public health resource allocation. An HbA1c difference of approximately 0.02% over 2 years in individuals with FH_ DM translates to roughly 0.1% over 10 years. This projection, however, assumes a constant rate of progression, which may not hold over longer periods due to regression to the mean, lifestyle modifications, or pharmacological interventions. Longer-term studies are therefore needed to confirm whether the observed differences persist and accumulate over time. Given that the threshold for diabetes diagnosis is HbA1c > 6.5% and for prediabetes is 5.7–6.4% [7], this accelerated progression could shift individuals across diagnostic categories several years earlier than those without FH_ DM. The pattern for FBG was even more pronounced, with differences increasing from approximately 0.3 mg/dL over 2 years to 1.3–1.9 mg/dL over 4 years, suggesting that individuals with FH_ DM may reach prediabetic or diabetic FBG levels substantially earlier [6,7].

Our findings align with and extend previous epidemiological evidence. The Framingham Offspring Study demonstrated that family history nearly doubled the risk of type 2 diabetes [30,31], while other studies have documented associations between FH_ DM and various metabolic abnormalities [2–6]. However, most prior research focused on diabetes incidence as a binary outcome. By examining continuous biomarkers and their magnitude of change over time, our study captures earlier metabolic deterioration that precedes clinical diagnosis. Our findings also complement recent work by Plattner et al. [10], who examined post-diagnosis complications, whereas we investigated pre-diagnostic glycemic progression, together suggesting that the association of family history spans the entire diabetes natural history.

These findings have direct relevance for diabetes screening policies. Current guidelines in many countries, including South Korea, recommend routine diabetes screening every 3 years for average-risk adults [1]. However, our results suggest that individuals with FH_ DM may benefit from more frequent monitoring, such as annual or biennial glucose testing. Such a risk-stratified approach enables earlier detection when interventions are most effective, using a readily available risk marker that requires no additional testing, and may prove cost-effective by focusing intensive monitoring on high-risk individuals [3]. In South Korea, where standardized health checkups are widely available, systematically utilizing family history information could enhance diabetes prevention efforts at the population level.

The consistency of results across IPTW and doubly robust approaches strengthens confidence in our findings, with the doubly robust method providing additional protection against model misspecification [25,26]. In the 4-year analysis, PSM produced larger estimates than IPTW and DR, likely because matching restricts the analysis to individuals with sufficient covariate overlap and thereby changes the analytic population. Thus, the PSM estimates may reflect effects in the matched subset rather than the overall study population. We therefore interpreted the PSM results as supportive sensitivity evidence and used the IPTW and DR estimates as the primary reference for the magnitude of association. Sensitivity analyses using robustness values indicated that unmeasured confounders would need to explain 1.1–4.4% of residual variance in both FH_ DM and the outcomes to nullify our findings, suggesting caution in interpretation. Nevertheless, the comprehensive adjustment for 49 measured covariates and consistency across methods support the validity of our estimates.

The stratified analysis by baseline glycemic status further supported the pooled analysis. The association between FH_ DM and glycemic progression was most consistent among individuals with normoglycemia or prediabetes, whereas estimates among those with diabetes were unstable because of limited sample size, particularly in the 4-year analysis, and may have been influenced by more intensive monitoring, lifestyle modification, or pharmacological management after diabetes recognition.

Several limitations warrant consideration. First, family history was self-reported and treated as a binary variable. The health checkup questionnaire did not distinguish between first-degree and more distant relatives, and underreporting would likely attenuate our estimates toward the null. Second, residual confounding from unmeasured factors such as diet, physical activity, and genetic polymorphisms remains possible, all of which are known to influence glucose metabolism [32]. Third, our study population was drawn from a single hospital’s health checkup program in Korea, which may limit generalizability to other populations. Fourth, the observational design cannot definitively establish causality, as family history reflects both genetic predisposition and shared environmental factors [2–5]. Finally, follow-up periods of 2 and 4 years are relatively short, and longer-term studies are needed to assess whether the observed differences ultimately translate to earlier diabetes onset.

Future research should incorporate more detailed family history information, lifestyle and genetic data, and multi-center designs across diverse populations to address these limitations and confirm the long-term implications of our findings.

## Conclusions

Using rigorous propensity score methods applied to longitudinal health checkup data, we found that family history of diabetes is associated with greater glycemic progression. Individuals with FH_ DM showed consistently larger increases in HbA1c and fasting blood glucose across multiple analytical approaches.

These findings support the use of FH_ DM as a practical risk marker for identifying individuals who may benefit from intensified monitoring and earlier preventive interventions. Given that family history information is readily obtained during routine health checkups without additional cost, integrating FH_ DM into risk-stratified screening strategies represents an actionable approach to diabetes prevention.

As diabetes prevalence continues rising globally, optimizing screening and prevention strategies for high-risk populations is increasingly important. Our results contribute evidence that can inform clinical guidelines and public health policies, ultimately supporting efforts to reduce the burden of diabetes through earlier detection and intervention.

## Supporting information

S1 File Anonymized dataset for Period 1 (2-year interval) and Period 2 (4-year interval). (ZIP)

S1 Table Stratified sensitivity analysis of the adjusted association of family history of diabetes with glycemic progression by baseline glycemic status. Values represent the difference in change in HbA1c (%) or FBG (mg/dL) between individuals with family history of diabetes and those without, with 95% confidence intervals in parentheses. Glycemic status was classified according to American Diabetes Association criteria. n, sample size (FH_DM / non-FH_DM); PSM, propensity score matching; IPTW, inverse probability of treatment weighting; DR, doubly robust estimation.
(PDF)
