# Supplementary material for: Family history of diabetes and glycemic progression: A propensity score-based analysis using health checkup data
Source: PLoS One. 2026 Jun 24;21(6):e0352348. doi: 10.1371/journal.pone.0352348 (PMC13293398; doi:10.1371/journal.pone.0352348)
Supplement: S1 Table — Values represent the difference in change in HbA1c (%) or FBG (mg/dL) between individuals with family history of diabetes and those without, with 95% confidence intervals in parentheses. Glycemic status was classified according to American Diabetes Association criteria. n, sample size (FH_DM / non-FH_DM); PSM, propensity score matching; IPTW, inverse probability of treatment weighting; DR, doubly robust estimation. (PDF) [file pone.0352348.s002.pdf]

Table 1. Stratified sensitivity analysis of the adjusted association of family history of diabetes with glycemic progression by baseline glycemic status. Values represent the difference in change in HbA1c (%) or FBG (mg/dL) between individuals with family history of diabetes and those without, with 95% confidence intervals in parentheses. Glycemic status was classified according to American Diabetes Association criteria. n, sample size (FH_DM / non-FH_DM); PSM, propensity score matching; IPTW, inverse probability of treatment weighting; DR, doubly robust estimation.

**Normoglycemia (FBG $\leq$ 100 mg/dL and HbA1c $\leq$ 5.7%)**				
HbA1c (%)				
**Period**	n	**PSM**	**IPTW**	**DR**
1-period (2 years)	17,947	0.011 (0.006, 0.016)	0.019 (0.012, 0.026)	0.020 (0.009, 0.032)
2-period (4 years)	8,848	0.011 (0.004, 0.019)	0.010 (−0.001, 0.020)	0.012 (−0.004, 0.029)
FBG (mg/dL)				
**Period**	n	**PSM**	**IPTW**	**DR**
1-period (2 years)	17,947	0.394 (0.193, 0.594)	0.432 (0.151, 0.714)	0.428 (−0.026, 0.914)
2-period (4 years)	8,848	0.435 (0.144, 0.726)	0.763 (0.359, 1.167)	0.762 (0.123, 1.429)
**Prediabetes (FBG 100–125 mg/dL or HbA1c 5.7–6.4%)**				
HbA1c (%)				
**Period**	n	**PSM**	**IPTW**	**DR**
1-period (2 years)	7,237	0.025 (0.013, 0.038)	0.038 (0.020, 0.057)	0.039 (0.013, 0.065)
2-period (4 years)	3,809	0.054 (0.030, 0.079)	0.068 (0.033, 0.103)	0.073 (0.025, 0.127)
FBG (mg/dL)				
**Period**	n	**PSM**	**IPTW**	**DR**
1-period (2 years)	7,237	0.643 (0.237, 1.049)	0.582 (0.003, 1.160)	0.587 (−0.331, 1.467)
2-period (4 years)	3,809	1.328 (0.662, 1.994)	2.000 (1.041, 2.958)	2.010 (0.518, 3.470)
**Diabetes (FBG $\geq$ 126 mg/dL or HbA1c $\geq$ 6.5%)**				
HbA1c (%)				
**Period**	n	**PSM**	**IPTW**	**DR**
1-period (2 years)	322	0.003 (−0.175, 0.181)	0.064 (−0.176, 0.305)	0.072 (−0.565, 0.775)
2-period (4 years)	67	−0.518 (−0.860, −0.176)	−0.870 (−1.275, −0.464)	−0.359 (−1.336, 0.654)
FBG (mg/dL)				
**Period**	n	**PSM**	**IPTW**	**DR**
1-period (2 years)	322	−7.801 (−12.427, −3.175)	−6.995 (−13.192, −0.797)	−6.341 (−23.672, 11.602)
2-period (4 years)	67	−5.105 (−13.629, 3.420)	−4.392 (−13.093, 4.310)	−11.032 (−28.545, 29.597)
